# AT1b receptors contribute to regional disparities in angiotensin II mediated aortic remodelling in mice

**DOI:** 10.1098/rsif.2024.0110

**Published:** 2024-08-28

**Authors:** Cristina Cavinato, Bart Spronck, Alexander W. Caulk, Sae-Il Murtada, Jay D. Humphrey

**Affiliations:** ^1^ Department of Biomedical Engineering, Yale University, New Haven, CT, USA; ^2^ LMGC, Univ. Montpellier, CNRS, Montpellier, France; ^3^ Department of Biomedical Engineering, Cardiovascular Research Institute Maastricht (CARIM), Maastricht University, Maastricht, The Netherlands; ^4^ Vascular Biology and Therapeutics Program, Yale School of Medicine, New Haven, CT, USA

**Keywords:** aorta, hypertension, sex, stiffness, inflammation, remodelling

## Abstract

The renin–angiotensin system plays a key role in regulating blood pressure, which has motivated many investigations of associated mouse models of hypertensive arterial remodelling. Such studies typically focus on histological and cell biological changes, not wall mechanics. This study explores tissue-level ramifications of chronic angiotensin II infusion in wild-type (WT) and type 1b angiotensin II (AngII) receptor null (*Agtr1b* ^−/−^) mice. Biaxial biomechanical and immunohistological changes were quantified and compared in the thoracic and abdominal aorta in these mice following 14 and 28 days of angiotensin II infusion. Preliminary results showed that changes were largely independent of sex. Associated thickening and stiffening of the aortic wall in male mice differed significantly between thoracic and abdominal regions and between genotypes. Notwithstanding multiple biomechanical changes in both WT and *Agtr1b* ^−/−^ mice, AngII infusion caused distinctive wall thickening and inflammation in the descending thoracic aorta of WT, but not *Agtr1b* ^−/−^, mice. Our study underscores the importance of exploring differential roles of receptor-dependent angiotensin II signalling along the aorta and its influence on distinct cell types involved in regional histomechanical remodelling. Disrupting the AT1b receptor primarily affected inflammatory cell responses and smooth muscle contractility, suggesting potential therapeutic targets.

## 1. Introduction

Among other effects, hypertension associates with central artery stiffening—a well-established initiator and indicator of cardiovascular disease risk and all-cause mortality [1–3]. The renin–angiotensin system plays a central role in blood pressure control [4–6], thus motivating many studies of mouse models of hypertensive arterial remodelling induced by chronic infusion of angiotensin II (AngII) at different rates (often 200, 490 or 1000 ng kg^−1^ min^−1^). Copious results are thus available in the literature, with most focusing on histological and cell biological changes [7,8]. There is, in addition, a critical need to understand tissue-level consequences of the underlying molecular mechanisms, particularly associated changes in distensibility as well as wall stress, stiffness and stored energy. Because such metrics depend on the biaxial loading, they are best assessed *ex vivo* using appropriate biomechanical testing [9]. Among others, we have presented biaxial findings for AngII-induced hypertensive remodelling of the aorta in wild-type (WT), *Apoe* ^−/−^, and *Fbln5* ^−/−^ mice, focusing on central arterial stiffness and associated aortopathies [10–14].

Increased AngII can affect the aorta both indirectly, by vasoconstricting distal vessels and increasing mean arterial pressure and thus aortic wall stress, and directly, by stimulating complex intracellular signalling downstream of the two primary AngII receptors, AT1R and AT2R [15]. The density of these receptors varies along the length of the murine aorta, with lower density proximally and higher density distally [16,17]. AT1R exists as two isoforms in rodents, AT1aR and AT1bR [18], the former of which is thought to be the dominant mediator of hypertension and cardiovascular remodelling in adult mice. Aortic remodelling, including medial degeneration and adventitial fibrosis, often contributes to the progression of diverse cardiovascular diseases, including central artery stiffening and aortic aneurysms. Diverse roles of AT1aR have been studied in these and related vascular contexts using both global and cell-specific gene deletion (e.g. [16,19–26]). By contrast, many fewer studies have focused on AT1bR, although it has been suggested that AT1b, not AT1a, receptors are key mediators of local AngII-induced vasoconstriction [17,27,28], thus playing a role in modulating blood pressure [29]. Given that some aspects of AngII-induced aortic remodelling and disease can be triggered separate from blood pressure elevation [26,30,31], there is a need for more understanding of the biomechanical and physiological roles of both AT1aR and AT1bR, especially since understanding nuances between receptor sub-types could help us to interpret better the extensive literature on AngII-infusion in mice and possibly suggest new treatment strategies as current AT1R blockers target both AT1aR and AT1bR in mice without distinction [32].

We hypothesized that differential mechanical responses would emerge regionally in AngII-induced hypertension in mice with germline deletion of the gene (*Agtr1b*) that encodes AT1bR. Hence, in this paper, we contrast the effects of short-term (14 or 28 day) continuous infusions of AngII at 1000 ng kg^−1^ min^−1^ in adult WT and type 1b angiotensin II receptor null (*Agtr1b* ^−/−^) mice by comparing remodelling across three regions of the aorta, the ascending (ATA) and descending (DTA) thoracic aorta and the infrarenal abdominal aorta (IAA).

## 2. Results

Morphological assessments, immunohistological studies and passive and active biomechanical metrics obtained from cyclic *ex vivo* extension–distension tests were evaluated together to assess possible differences in AngII-induced remodelling along the length of the aorta. Morphological assessments included gross measurements of dimensions; immunohistological studies focused on the primary structural constituents within the medial and adventitial layers as well as immune cell infiltration; biaxial stretch and stress were inferred directly from experimental measurements while other mechanical metrics were computed using a best-fit constitutive relation for the passive behaviour based on nonlinear regression of the pressure-diameter and axial-force length data. Vasoconstriction and vasodilation were assessed *ex vivo* under fixed luminal pressure and axial stretch in response to multiple vasoactive substances. All data were evaluated for statistical significance. Finally, transmural stresses were calculated using validated methods [33]. Details of each of these procedures are in §4.

### 2.1. Primary effects of AngII are independent of sex in AT1bR null mice

We performed a preliminary study to determine if sex was a critical biological variable for our cohort of *Agtr1b* ^−/−^ mice. Passive and active biomechanical metrics and histology were compared across *n* = 20 mice at 14 weeks of age (= 12 weeks + 2 weeks of AngII infusion or not): 10 female and 10 male *Agtr1b* ^−/−^ mice, 5 each without or with 14 days of AngII infusion. These data, collected for both thoracic (DTA) and abdominal (IAA) aortic segments, revealed that baseline biomechanical properties were similar for adult female and male receptor null mice. Moreover, AngII-induced aortic remodelling was largely independent of sex in these mice, both in terms of biomechanical properties and histological composition (electronic supplementary material, figures S1 and S2). One difference was a significantly lower area fraction (AF) of adventitial collagen in female compared with male mice, with a related higher elastin fraction. Yet, there were no significant variations in the actual area of these components (data not shown). Hence, thereafter we focused on male WT and *Agtr1b* ^−/−^ mice, which could be compared better against the majority of data available in the literature.

Based on mechanical properties, aortas from male *Agtr1b* ^−/−^ mice demonstrated natural ageing up to 52 weeks of age (electronic supplementary material, figures S3 and S4). We focused, however, on mice from 10 to 14 weeks of age, which yields a biomechanically mature aorta [34] without confounding effects of ageing [35], also facilitating comparisons with the majority of data in the literature.

### 2.2. AngII-induced changes in aortic morphology and distensibility differ by region and genotype

**Figure 1.**
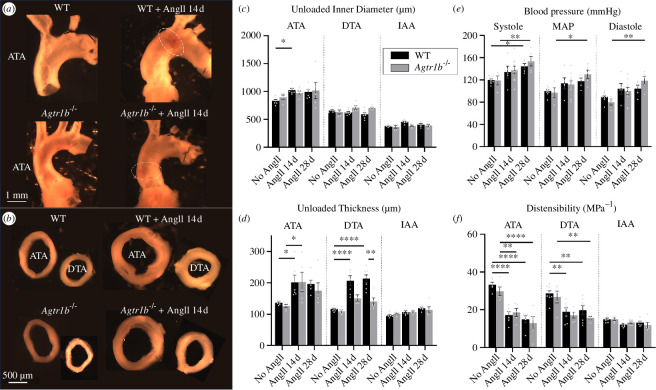
(*a*) Illustrative gross e*x vivo* morphometry of excised samples including the ATA and aortic arch and (*b*) transversely sectioned aortic rings for the ATA and DTA, both for male WT and *Agtr1b* ^−/−^ mice without (no AngII) and with (AngII) 14 day infusions of angiotensin II. The dashed white ellipse in (*a*) shows regions of intramural haematoma. These selected segments exhibited the most significant differences in unloaded (*c*) internal diameter and (*d*) wall thickness of the three analysed regions: ATA, DTA and IAA. Infusion of AngII in WT and *Agtr1b* ^−/−^ mice induced a slight luminal enlargement of the ATA, significant in WT but not in *Agtr1b* ^−/−^, but unremarkable changes in other regions. AngII also induced wall thickening in the ATA and DTA in WT mice and in the ATA but not DTA in *Agtr1b* ^−/−^ mice; changes were mild in the IAA of both groups. (*e*) Blood pressures (systolic, mean arterial pressure and diastolic) increased progressively with the duration of AngII infusion independent of genotype. (*f*) *Ex vivo* testing revealed marked decreases in computed distensibility in the ATA, less in the DTA and little in the IAA. These effects of exogenous AngII were evident after 14 days of infusion, with few changes thereafter to 28 days. Sample sizes: *n* = 6 for ATAs of all WT mice, *n* = 9 for DTAs of WT mice without AngII and *n* = 7 for DTAs of WT mice with AngII for 14 and 28 days, *n* = 7 for IAAs of WT mice without with 14 days infusions of AngII and *n* = 5 for IAAs of WT mice with 28 days infusions of AngII; *n* = 5 for all samples of *Agtr1b* ^−/−^ mice. *, **, *** and **** indicate statistical significance at *p* < 0.05, 0.01, 0.001 and 0.0001, respectively. Explicit numerical comparisons can be found in electronic supplementary material, tables S1–S3.

Unloaded dimensions of aortic cross-sections were compared at three locations (ATA, DTA and IAA) for 14-week-old male WT and *Agtr1b* ^*−/−*^ mice having been exposed to 0 (i.e. normotensive controls), 14 or 28 days of AngII infusion. These gross morphological measurements revealed modest AngII-induced changes in unloaded radius but marked thickening of the ATA without thickening of the IAA in both genotypes (figure 1*a*–*d*). Despite insignificant differences in blood pressure elevation between AngII-infused WT and *Agtr1b* ^*−/−*^ mice (figure 1*e*), there was marked thickening of the wall in the DTA in WT but not *Agtr1b* ^*−/−*^ mice (figure 1*b,d*). These gross differences revealed a need for a careful biomechanical phenotyping and histological examinations across regions and genotypes.

### 2.3. Passive biomechanical properties differ by genotype, region and layer

**Figure 2.**
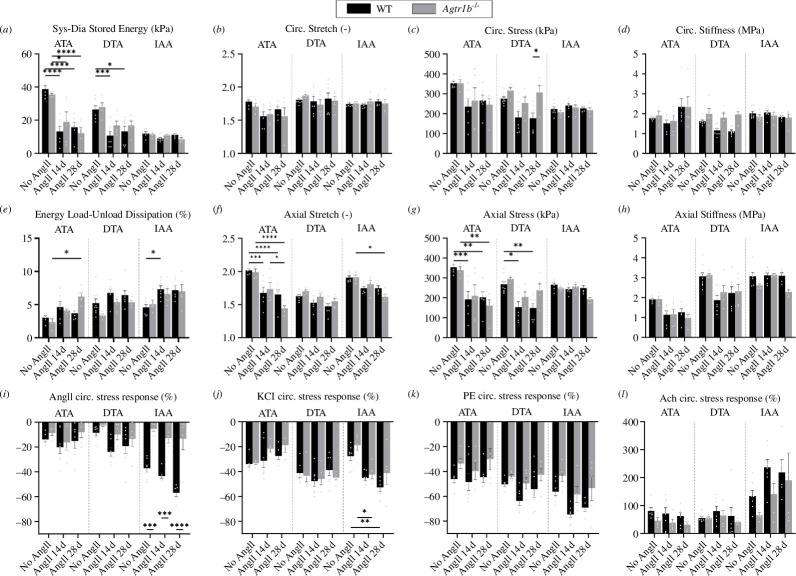
*Ex vivo* mechanical characterization of the ATA, DTA and IAA from male WT and *Agtr1b* ^−/−^ mice. (*a,e*) Chronic AngII infusion tended to reduce cyclically stored elastic energy (between group-specific systolic and diastolic pressures) in the ATA and DTA but not in the IAA while generally increasing dissipation (over the entire tested pressure interval, from 10 to 140 mmHg). (*b,f*) Circumferential (Circ) and especially axial stretch decreased in the ATA with AngII infusion, and axial stretch showed the same decreasing trend in the DTA. (*c,g*) Circumferential and axial Cauchy stresses were also reduced in the ATA but not in the IAA with AngII infusion; by contrast, AngII-induced reductions in stress in the DTA were restricted to the WT aortas. (*d,h*) Similar findings held for biaxial material stiffness, and axial stiffness in particular, with the ATA most affected and IAA least affected while the DTA showed genotype-specific differences. (*i–l*) *Ex vivo* vasoactive responses to 10 μM AngII (followed by wash-out), 100 mM KCl (followed by wash-out), 1 μM PE, then 10 µm ACh while pressurized at 90 mmHg and held at the specimen-specific *in vivo* axial stretch. Responses are shown as per cent changes in calculated circumferential wall stress owing to vasoactive substance-mediated changes in luminal diameter and wall thickness; a 0% change would indicate no effect of the vasoactive substance. Note (*i*) the reduced AngII-mediated responses in the control *Agtr1b^−/−^* aortas as expected, especially in the IAA. These control *Agtr1b* ^−/−^ aortas were yet able to vasoconstrict well (*j,k*) when exposed to KCl and, to a slightly lesser extent, to PE. AngII-mediated changes were greater in all three regions in both WT and KO following *in vivo* AngII infusion for 14 days. This response was particularly marked in WT IAA, which increased further after 28 days of *in vivo* AngII infusion. KCl-mediated responses tended to decrease slightly in the ATA, stabilize in the DTA, and increase significantly in the IAA after 14 and 28 day AngII treatment, with no clear difference between WT and *Agtr1b^−/−^*. PE-mediated responses showed a mild increase in WT aortas with 14 days of AngII infusion followed by a slight decrease after 28 days of infusion, with slightly greater changes in more distal regions. The contractile response to PE was generally, but not significantly, attenuated in *Agtr1b* ^−/−^ aortas, also after AngII treatment for 14 and 28 days. The (*l*) EC-dependent relaxation response to ACh was highest in the IAA (commensurate with the highest pre-constriction with PE) which tended to increase after 14 days of AngII infusion and stabilize afterward. ACh-mediated responses were slightly reduced in *Agtr1b* ^−/−^ samples in all regions and with AngII infusions. Sample sizes: *n* = 6 for ATAs of all WT mice, *n* = 9 for DTAs of WT mice without AngII and *n* = 7 for DTAs of WT mice with AngII for 14 and 28 days, *n* = 7 for IAAs of WT mice without with 14 days infusions of AngII and *n* = 5 for IAAs of WT mice with 28 days infusions of AngII; *n* = 5 for all samples of *Agtr1b* ^−/−^ mice. *, **, *** and **** indicate statistical significance at *p* < 0.05, 0.01, 0.001 and 0.0001, respectively. Explicit numerical comparisons can be found in electronic supplementary material, tables S1–S3. Per cent changes mechanical metrics are reported in electronic supplementary material, figure S5*c–p* in the case of no AngII infusion and AngII infusion for 14 days. KCl, potassium chloride; PE, phenylephrine; ACh, acetylcholine.

Biaxial testing of passive aortic segments across these same three regions (ATA, DTA and IAA) from 14-week-old male WT and *Agtr1b* ^−/−^ mice having AngII infusion for 0, 14 or 28 days similarly revealed complex differences. Distensibility, a measure of structural stiffness, decreased the most in the ATA, less so in the DTA, and little in the IAA independent of genotype after 14 and 28 days of AngII infusion (figure 1*f*). Findings were similar for the experimentally inferred, energetically preferred value of axial stretch and computed values of cyclic elastic energy storage and axial material stiffness (figure 2*a,f,h*). Findings for the experimentally inferred circumferential stretch were similar, but less distinct (figure 2*b*). By contrast, experimentally inferred biaxial wall stresses (figure 2*c,g*) and computed circumferential material stiffness (figure 2*d*) revealed marked decreases within the DTA of AngII-infused WT but not *Agtr1b* ^−/−^ mice, consistent with genotype-specific regional differences in wall thickening. Computed dissipation of energy was generally higher following AngII infusion independent of genotype, though less marked in the DTA in the absence of *Agtr1b* (figure 2*e*). See also electronic supplementary material, figure S5.

### 2.4. Active biomechanical properties differ by region and genotype

Vasoactivity can affect the state of stress of the wall of a pressure-distended aorta. *Ex vivo* vasoconstriction in response to 10 μM AngII in the adventitial bath was less in the thoracic than in the abdominal aorta in WT mice, as expected based on normal distributions of AT1 receptors, and this response was blunted in all aortic regions in *Agtr1b* ^−/−^ mice, thus confirming functional consequences of the global germline knockout (figure 2*i*). Responses to 100 mM potassium chloride (KCl) in the adventitial bath yet revealed that the smooth muscle cells were capable of contracting in the aorta of *Agtr1b* ^−/−^ mice similar to the aorta in WT mice (figure 2*j*); responses to 1 μM phenylephrine (PE) were blunted, however, in all regions of the *Agtr1b* ^−/−^ aorta relative to WT (figure 2*k*), which was paralleled by levels of acetylcholine-induced (10 μM in the adventitial bath) vasodilation following pre-constriction with PE (figure 2*l*). There appears, therefore, to be some cross-talk between AngII- and PE-induced contraction of smooth muscle.

### 2.5. Histological characteristics and inflammatory cell infiltration differ by region and genotype

**Figure 3.**
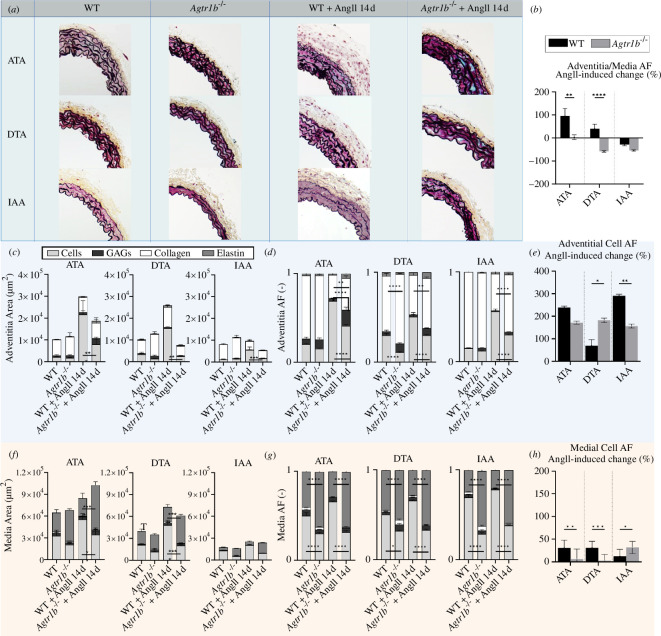
Histological characterization. Area (µm^2^) and AFs (area fractions, dimensionless) of cells, GAGs (glycosaminoglycans), collagen, and elastin were computed from (*a*) images of Movat Pentachrome staining via colorimetric analysis in (*c,d*) adventitial and (*f,g*) medial layers of WT (first, third columns) and *Agtr1b* ^−/−^ (second, fourth columns) aortas, without and with AngII infusion for 14 days in all three aortic regions (ATA, DTA and IAA). Adventitial and medial layer areas were used to compute (*b*) the relative per cent change in adventitia/media AF in response to 14 days of AngII infusion. Cell AF in the (*e*) adventitia and (*h*) media were also described as per cent changes in response to 14 days of AngII infusion. AngII induced a significant increase in cell AF in both layers and related decreases in collagen AF in the adventitia and elastin AF in the media in all regions in both WT and *Agtr1b* ^−/−^ aortas. The *Agtr1b* deletion induced a decreased cell AF in the entire wall, along with an associated increase of collagen AF and GAG AF in the adventitia and elastin in the media. AngII induced an increase in the adventitia : media ratio in the WT ATA and DTA, with no change in the *Agtr1b* ^−/−^ ATA, a reduction in *Agtr1b* ^−/−^ DTA and a reduction in both groups in the IAA. Nonetheless, a clear reduction in the overall area of adventitial and medial cells was observed in *Agtr1b* ^−/−^ compared with WT after AngII infusion in all regions, particularly in the thoracic regions. Sample sizes: *n* = 6 for ATAs of all WT mice, *n* = 9 for DTAs of WT mice without AngII and *n* = 7 for DTAs of WT mice with AngII for 14 and 28 days; *n* = 7 for IAAs of WT mice without with 14 days infusions of AngII and *n* = 5 for IAAs of WT mice with 28 days infusions of AngII; *n* = 5 for all samples of *Agtr1b* ^−/−^ mice. *, **, *** and **** indicate statistical significance at *p* < 0.05, 0.01, 0.001 and 0.0001, respectively.

Despite the progressive increases in blood pressure from 0 to 14 to 28 days of AngII infusion (figure 1*e*), changes in aortic morphology, distensibility and other biomechanical metrics were generally similar after 14 and 28 days of infusion (figures 1 and 2), hence we focused our histological and immunohistochemical analyses across the three regions (ATA, DTA and IAA) for the 0 and 14 day infusion groups. Histology confirmed wall thickening following AngII infusion, especially within the thoracic segments (figure 3*a*). This thickening was preferentially adventitial in the ATA and DTA in WT mice, but medial in the DTA and IAA in *Agtr1b* ^−/−^ mice (figure 3*b*). Notwithstanding changes in the AFs of extracellular matrix in these two primary layers of the wall, there were also marked changes in the percentage of cells, particularly within the adventitia in the thoracic aorta. Note, however, the attenuated AngII-induced increase in cellular area and cell AF in both layers in the *Agtr1b* ^−/−^ aortas (figure 3*c–h*).

**Figure 4.**
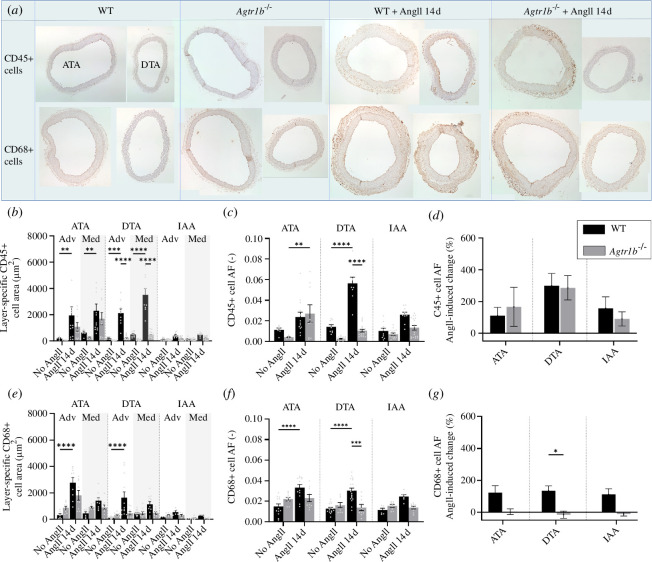
Inflammatory cell classification. Images of (*a*, first row) CD45-positive cells (i.e. leucocytes) and (*a*, second row) CD68-positive cells (i.e. macrophages) in the ATA (left) and DTA (right) of WT and *Agtr1b* ^*−/−*^ mice without and with infusion with AngII for 14 days. Colorimetric analysis was used to quantify (*b, e*) absolute area, (*c, f*) AFs (area fractions) and (*d, g*) per cent changes without and with 14 days of AngII infusion in the ATA, DTA and IAA. AngII infusion increased CD45+ cell AF in both WT and *Agtr1b* ^*−/−*^ aortas. Yet, in terms of absolute area, the increase was lower in the *Agtr1b* ^*−/−*^ ATA than the WT ATA, not observed in the *Agtr1b* ^*−/−*^ DTA but prominent in the WT DTA, and minimal in IAA of both groups. AngII infusion increased CD68+ cells only in WT. The DTA in *Agtr1b* ^*−/−*^ mice infused with AngII had significantly fewer CD68+ cells than the DTA in WT mice, and the per cent change in CD45+ cell AF was less in this region than in others. See electronic supplementary material, figure S6 for quantification of CD3-positive cells, that is T-cells. Sample sizes: *n* = 6 for ATAs of all WT mice, *n* = 9 for DTAs of WT mice without AngII and *n* = 7 for DTAs of WT mice with AngII for 14 and 28 days, *n* = 7 for IAAs of WT mice without with 14 days infusions of AngII and *n* = 5 for IAAs of WT mice with 28 days infusions of AngII; *n* = 5 for all samples of *Agtr1b* ^−/−^ mice.

Immunohistochemical staining for CD45+ leukocytes and CD68+ macrophages revealed a marked increase in immune cells in the ATA of both AngII-infused WT and *Agtr1b* ^−/−^ mice (figure 4*a–c*). By contrast, a significant augmentation of marker area in the DTA was seen in the AngII-infused WT, not the *Agtr1b* ^−/−^, mice. It should be noted that the number of CD45+ cells started at lower levels in the aortas of control *Agtr1b* ^−/−^ mice compared with control WT mice, thus the percentage change in leukocyte AF was similar between the two groups (figure 4*d*). There was also an increase in the area of the CD68 macrophage marker in the aorta of AngII-infused WT mice, particularly in thoracic regions. On the other hand, *Agtr1b* ^−/−^ data did not exhibit a clear increase in macrophage AF following AngII infusion in any region (figure 4*e–g*). CD3+ staining showed slight but non-significant increases in T-cell area in the ATA and DTA in WT mice exposed to AngII infusion, but the T-cell AF generally decreased in all regions and both genotypes after infusion (electronic supplementary material, figure S6).

### 2.6. Bi-layered stress analysis suggests contrasting effects on distal thoracic arterial wall mechanics

**Figure 5.**
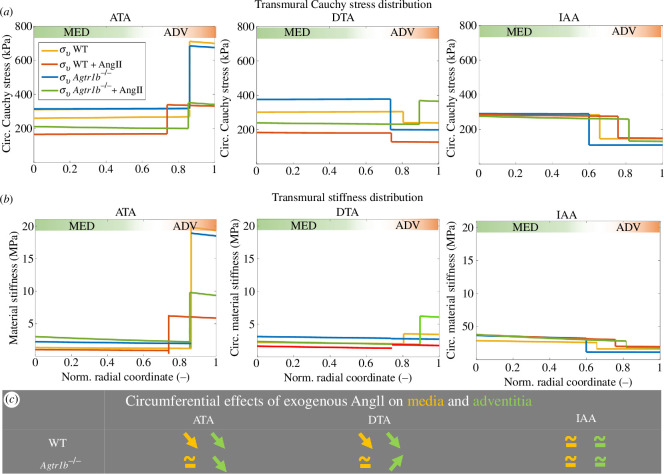
Calculated transmural distributions of circumferential (*a*) Cauchy wall stress and (*b*) material stiffness presented as group averages for the ATA (left), DTA (middle) and IAA (right) in WT and *Agtr1b* ^*−/−*^ mice, each at genotype-specific systolic pressure and group-specific experimentally inferred *in vivo* (i.e. energetically preferred) axial stretch. The only cells considered to be load-bearing were the smooth muscle cells of the media, assumed to be oriented circumferentially. Focusing on the two primary layers separately (*c*) (yellow and green arrows indicate trends in media and adventitia, respectively), stress and stiffness in the circumferential direction tended to decrease in both the adventitia and media in the ATA following 14 days of AngII-induced pressure elevation, whereas they tended to remain constant in both layers in the IAA. In the DTA, the sustained AngII-induced pressure elevation led to different distributions in WT and *Agtr1b* ^*−/−*^ mice. In WT, DTA stress and stiffness tended to decrease in the adventitia and media (due in part to excessive thickening), while in *Agtr1b* ^*−/−*^, DTA stress and stiffness increased in the adventitia (due in part to a lack of adequate thickening). In contrast with WT, the adventitia in AngII-infused *Agtr1b* ^*−/−*^ DTAs was generally bearing more load than the media.

Given the measured layer-specific differences in AngII-induced changes in wall thickness and cell content, we next used a bi-layered equilibrium analysis to compute medial and adventitial mechanics using layer-specific anisotropic constitutive relations determined from our data [14,33]. We prescribed measured differences in histological composition and the ratio of adventitial: medial areas to estimate AngII-induced changes in transmural wall stress and material stiffness in circumferential (figure 5*a,b*) and axial (electronic supplementary material, figure S7*a*,*b*) directions based on group-specific systolic pressures. Inflammatory and adventitial cells were assumed to have no principal orientation and were considered non-loadbearing. After infusion of AngII, both circumferential and axial stress and stiffness decreased in the adventitial and medial layers of the ATA owing to excessive thickening of the wall. Conversely, in the IAA, there were no significant changes as a result of AngII infusion, except for a slight increase in axial stress and stiffness in the adventitia of the IAA in *Agtr1b* ^−/−^ mice.

By contrast, simulated effects for the DTA differed distinctly between WT and *Agtr1b* ^−/−^ mice. Following a simulated elevation in systolic blood pressure and reduction in the *in vivo* value of axial stretch associated with AngII infusion, both adventitial and medial wall stress and stiffness tended to decrease in the circumferential and axial directions in the WT DTA. Conversely, in the *Agtr1b* ^−/−^ DTA, wall stress and stiffness increased in the adventitia but stabilized in the media with AngII infusion (figure 5*c*). All fitted parameters of the bi-layered model computed for each group are reported in electronic supplementary material, table S4.

### 2.7. Correlations

**Figure 6.**
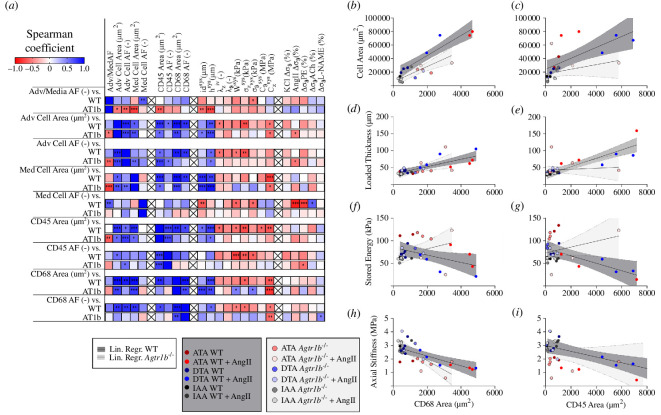
Non-parametric Spearman correlations among cell areas and AFs (area fractions) and passive and active mechanical metrics. (*a*) The correlation matrix was computed across adventitia : media ratio, cell areas and AFs in the adventitia and media, CD45+ and CD68+ cell areas and AFs, and mechanical parameters (found in prior figures), the latter calculated at systolic pressure when specified with a corresponding superscript. To identify genotype-related differences, correlation coefficients were computed separately for WT and *Agtr1b* ^−/−^ samples with no distinction by region. (*b–i*) Selected relationships showing significant linear correlation for both WT and *Agtr1b* ^−/−^ groups or for WT only, demonstrating differences in roles of inflammatory contributions via leukocyte and macrophage infiltration. Relationships exhibited similar positive correlations between increases in both CD45+ and CD68+ cell area and increases in total cell area as well as increases in wall thickness. Additionally, there was a correlation between the decreasing axial stiffness and the increasing area of CD45+ and CD68+ cells in both WT and *Agtr1b* ^−/−^ groups. The stored energy decreased significantly with increasing area of CD45+ and CD68+ cells in WT mice, but not in *Agtr1b* ^−/−^ mice. The correlation analysis was carried out also for all WT and *Agtr1b* ^−/−^ with distinction by region (electronic supplementary material, figures S8 and S9).

Given the complexity of the data—multiple geometric, biomechanical and histological markers across three segments of the aorta in two genotypes without or with AngII infusion—we computed unbiased correlations to seek potential relationships (figure 6*a*, electronic supplementary materials, S8, S9). Among the many correlations that emerged, consider those relating changes in total cell density with infiltrating inflammatory cells (CD45+ and CD68+) that were significatively positive for both WT and *Agtr1b* ^−/−^ mice (figure 6*b,c*). Moreover, hypertension-induced changes in geometric (wall thickness) and mechanical (axial stiffness) metrics also correlated well with CD45+ and CD68+ cells for both WT and *Agtr1b* ^−/−^ (figure 6*d,e*) mice. On the other hand, infiltrating inflammatory cells exhibited different correlations with stored energy at systole between WT and *Agtr1b* ^−/−^, with the former showing a significant negative correlation in terms of CD68+ cell area and the latter showing significant positive correlation in terms of CD45+ cell area. This finding is consistent with different interactions between the distinct immune cells in WT and *Agtr1b* ^−/−^ mice, resulting in localized morphometric differences in terms of thickness and the observed divergent mechanical responses.

## 3. Discussion

Vascular cells exhibit substantial transcriptional changes in hypertension that drive associated remodelling of the arterial wall. This remodelling can encompass progressive development of a severe biomechanical phenotype evidenced, in part, by either an accumulation or degradation of extracellular matrix, decreased smooth muscle contractility and increased inflammation [12,14,26]. Importantly, in response to AngII-induced hypertension, such remodelling tends to vary dramatically by region within the murine aorta, with the ascending thoracic aorta susceptible to dilatation, the descending thoracic aorta susceptible to fibrosis, the suprarenal abdominal aorta susceptible to intimo-medial damage and contained rupture and the infrarenal abdominal aorta susceptible to modest remodelling [11]. To avoid the extreme case of intimo-medial damage, we focused on the ATA, DTA and IAA. As expected, given the role of AT1bR in aortic contractility and its distribution along the aorta [17,27,28], we found that global germline knockout of *Agtr1b* markedly reduced the ability of the IAA to vasoconstrict *ex vivo* in response to transient AngII stimulation, which suggests that its ability to contract *in vivo* in response to chronic infusion of AngII was similarly compromised. Nevertheless, the IAA did not experience the adverse hypertensive remodelling that is characteristic of the less AngII-sensitive thoracic aorta in AngII-induced hypertensive WT mice. Rather adverse remodelling was seen in the ATA in both WT and *Agtr1b* ^−/−^ mice and in the DTA in WT, but not in *Agtr1b* ^−/−^, mice.

There are multiple possible reasons why knockout of *Agtr1b* may not have led to adverse hypertensive remodelling of the IAA. Reduced contractility may render a vessel more susceptible to hypertensive remodelling [12], but the IAA retained its ability to contract in response to both high KCl and phenylephrine. Alternatively, adverse aortic remodelling may be tied more to region-specific propensities to inflammatory cell infiltration following AngII infusion. This infiltration appeared to be most marked in the thoracic aorta and less pronounced in the infrarenal aorta. The central role of vascular inflammation in hypertension is clear [36,37], with infiltration of inflammatory cells into the aorta being a prominent characteristic, especially in AngII infusion models. For example, studies employing genetically modified mouse models and adoptive transfer of immune cells consistently demonstrate adverse roles of immune cells: deletion of the CCR2 monocyte receptor [38,39], deficiency of T- and B-cells [40,41], and increased immunosuppressive regulatory T-cells [42,43] all show protective effects against AngII-induced hypertension. Infusion of AngII in mice with a severe depletion of monocytes is also characterized by attenuated atherosclerosis [44] and thoracic aortic aneurysm [45]. Aortas in the former case also displayed an enhanced contractile response to phenylephrine in thin rings of the upper descending thoracic aortas, while no differences in contractile response were noted in the lower descending thoracic aorta when compared with control mice.

Although much has been discovered in recent years regarding roles of inflammatory cells in hypertensive arterial remodelling, less is known about their regionalized involvement in the aorta. An important finding of this study is that global deletion of the AT1b receptor resulted in significantly less adverse remodelling in the DTA. This distinct finding contributed, in large part, to the strong correlation that emerged between adverse remodelling—particularly overthickening of the wall and loss of elastic energy storage—and inflammatory cell infiltration. Moreover, much of this adverse remodelling manifested in the adventitia, where most of the inflammatory cells resided. Hence, the present results are consistent with prior studies showing, in mice having a C57BL/6 background, that both T-cells and macrophages contribute to the excessive adventitial remodelling of the thoracic aorta in AngII-induced hypertension [13,14,41,46]. The present results suggest further that the type 1b AngII receptor plays a key role in this inflammatory cell involvement.

The role of *Agtr1b* as a pro-inflammatory mediator has not been previously demonstrated in the aorta, but it has been confirmed in a mouse model of multiple sclerosis through induced autoimmune inflammatory disease of the central nervous system [47]. In this multiple sclerosis model, expression of *Agtr1b* was increased in macrophages, dendritic cells and T cells. Induction of this disease in *Agtr1a* ^−/−^ mice still resulted in severe disease that was prevented by inhibiting the residual receptor with losartan, thus highlighting an immunomodulatory and pro-inflammatory role of AT1b receptor.

Notwithstanding multiple novel findings, our study is not without limitations. We focused only on the aorta, noting that effects are probably different in other arteries, particularly muscular [34]. Comparing elastic versus muscular arteries was beyond the present scope. We did not quantify transcriptional changes, which can be revealing [14]. We performed a preliminary study evaluating possible effects of sex, but more detailed studies in female mice are warranted. We studied mice having a global germline knockout of the AT1b receptor. Studies using conditional knockout mice, which can avoid development changes owing to the knockout, and mice with cell-specific knockouts (cf. [22,24,26,48]). would similarly be informative, but we submit that the reported regional differences along the aorta and associated correlations herein are important nonetheless, particularly with regard to the many prior studies using AngII infusion in mice though without direct translation to the human. Furthermore, although the role of the AT1a receptor is generally more studied than that of the AT1b receptor due both to its more dominant mediation of hypertensive remodelling of the murine aorta in response to Ang II and its greater similarity to the AT1R in humans, regionalized biomechanical and histological data such as those presented here are not yet available for *Agtr1a* ^*−/−*^ mice. Such data will be necessary to compare roles of these two murine receptor sub-types and would help to unravel the intricate interplay between mechanics and cellular effects driven by AngII, which contribute to the development of pathological conditions. The same applies to AT2 receptors, which have an unclear role but are generally thought to counteract the signalling and functions of AT1 receptors as the loss of AT2 expression accelerates the aberrant growth and rupture of the aorta in prone mice [49,50].

In summary, AngII-induced hypertension in mice can stimulate adverse remodelling of the thoracic aorta, thus affecting many geometric, histological and biomechanical metrics that define its structure and function. Disruption of the AT1b receptor resulted in differential effects along the aorta, surprisingly singling out the descending thoracic aorta wherein the otherwise adverse AngII-induced hypertensive remodelling was attenuated significantly. It appears that the multiple roles of the AT1b receptor manifested more via its effects on immuno-modulation than smooth muscle cell vasoactivity. There remains a need, however, for continued study of the diverse actions of excessive exogenous AngII both in elevating blood pressure and differentially affecting different cell types that contribute to the regional variations in aortic remodelling.

## 4. Methods

### 4.1. Animals and specimen preparation

All live animal procedures were approved by the Institutional Animal Care and Use Committee of Yale University. Wild-type C57BL/6J (WT) mice were purchased from Jackson Laboratory and mutant knockout (*Agtr1b* ^−/−^) mice were generated by mating heterozygous (*Agtr1b^+/^* ^−^) mice. Young adult (14 weeks of age) female and male mice and aged (52 weeks of age) male mice were studied. Consistent with ARRIVE Guidelines, randomly selected age-matched mice underwent subcutaneous implantation of osmotic mini-pumps (Alzet model #2004, DURECT Corporation, Cupertino, CA, USA) for infusion of AngII at a rate of 1000 ng kg^−1^ min^−1^ for either 14 or 28 days. Subcutaneous implantation of the pumps was on the flank while the mice were anaesthetized with isoflurane (3% for induction, 1.5% for maintenance); buprenorphine (0.1 mg kg^−1^ subcutaneously) was given pre- and post-operatively for analgesia. *In vivo* blood pressure was measured using a Coda™ tail cuff system (Kent Scientific, Torrington, CT, USA) on mice upon reaching the indicated age, ensuring consistent daily time and temperature conditions. Subsequently, the mice were euthanized with an intraperitoneal injection of Beuthanasia-D, and three aortic regional segments were gently excised and prepared for biomechanical phenotyping: ATA, DTA and IAA. In all cases, death was confirmed by loss of cardiovascular function following exsanguination.

### 4.2. Mechanical testing

Using a custom computer-controlled experimental biaxial testing system [9] and previously established protocols [14], vessels were mounted on glass cannulae and secured at each end with 6–0 silk sutures within an oxygenated, buffered Krebs–Ringer bicarbonate buffered solution containing 2.5 mM CaCl_2_ at 37°C. After axially stretching the vessel to its specimen-specific *in vivo* length and pressurizing it to 90 mmHg, cell vasoactive responses were assessed as diameter changes during 10 min exposures to 10 μM AngII in the adventitial bath (followed by wash-out with normal Krebs), then 100 mM KCl to depolarize the cell membrane (followed by wash-out with normal Krebs), then 1 μM phenylephrine to activate the α1-adrenergic receptor and then 10 μM acetylcholine to stimulate endothelial-dependent release of nitric oxide. Outer diameter, pressure, length and axial force were measured under the isometric–isobaric constraints and wall thickness was calculated assuming incompressibility. Subsequently, following well-established protocols [11,51], the Krebs–Ringer solution was replaced with a standard Hank’s buffered physiologic solution at room temperature to minimize smooth muscle contractility and thereby focus on passive biomechanical properties. Vessels were then pre-conditioned via four cycles of pressurization while length was held fixed at specimen-specific *in vivo* values. Next, the vessels were subjected to three pressure–diameter (*P*–*d*) protocols, with luminal pressure cycled from 10 to 140 mmHg while axial stretch was maintained fixed at the specimen-specific *in vivo* value or 95% and 105% of this value. Finally, four axial force–length (*f*–*l*) protocols, with force cycled between 0 and a value equal to its maximum measured during the pressurization test at 5% above the *in vivo* axial stretch, were performed with luminal pressure maintained fixed at 10, 60, 100 or 140 mmHg for each segment ATA, DTA and IAA. Distending pressure, applied axial force, outer diameter and axial length were recorded online for all seven cyclic protocols, with more than 2800 data points collected per sample. Wall thickness was measured in the unloaded configuration using a dissecting microscope, which under the assumption of incompressibility allowed wall thickness to be calculated at each pressure–force state.

### 4.3. Uni-layered constitutive modelling

Multiple consistently calculated metrics collectively enable detailed biomechanical phenotyping of arteries across vessel types, sex, age and genotype [9]. These metrics include radially averaged intramural biaxial stress and stretch, material stiffness, elastic energy storage and dissipation during cyclic loading as well as distensibility. We used an independently validated [52] four-fibre family pseudostrain energy function W to quantify the passive behaviour, which can be written as


(4.1) $$W\left(C,M^{i}\right)=\frac{c}{2}\left(I_{C}-3\right)+\sum_{i=1}^{4}\frac{c_{1}^{i}}{4c_{2}^{i}}\left\{exp\left[c_{2}^{i}{\left(IV_{C}^{i}-1\right)}^{2}\right]-1\right\},$$

where c (kPa), $c_{1}^{i}$ (kPa) and $c_{2}^{i}$ (-) are material parameters ( i=1,2,3,4 denote four-fibre family directions), which are determined via nonlinear regression of *P*–*d* and *f*–*l* data from the last cycle of unloading in each of the seven protocols. Unloading data enable calculation of the non-dissipated (elastic) energy available to work on the luminal fluid. Finally, $I_{C}=trC$ and $IV_{C}^{i}\;=\;M^{i}\;\cdot \;CM^{i}$ are coordinate invariant measures of the finite deformation, with the right Cauchy–Green tensor $C=F^{T}F$ computed from the deformation gradient tensor $F=diag[\lambda_{r},\lambda_{\theta },\lambda_{z}],$ with detF=1 because of assumed incompressibility. The direction of the *i*th family of fibres is defined by $M^{i}=\left[0,\sin\alpha_{0}^{i},\cos\alpha_{0}^{i}\right]$ , with $\alpha_{0}^{i}$ denoting a fibre angle relative to the axial direction in the traction-free reference configuration. Based on prior micro-structural observations from multiphoton microscopy, and the yet-unquantified effects of cross-links among the multiple families of fibres, the four predominant families were axial ( $\alpha_{0}^{1}=0$ ), circumferential ( $\alpha_{0}^{2}=\pi /2$ ) and symmetric diagonal ( $\alpha_{0}^{3,4}={\pm \alpha }_{0}$ ). The value of $\alpha_{0}$ was included among the eight model parameters determined via the nonlinear regression. Values of transmurally averaged (mean) biaxial stress and stiffness were computed from the stored energy function and calculated at individually measured values of pressure and at a common pressure.

To investigate the impact of vasoconstriction on the biomechanical parameters mentioned above, we simulated active smooth muscle tone by considering that smooth muscle cells generate an active second Piola–Kirchhoff stress, which can be computed using the following equation:


(4.2) $$W^{m,act}\left(IV_{C}^{m}\right)=\;\frac{S^{act}}{2}\left(IV_{C}^{m}-1\right),$$

where $S^{act}$ represents an active stress parameter specific to the medial layer, and $IV_{C}^{i}\;=\;M^{m}\;\cdot \;CM^{m}$ are coordinate invariant measures of the finite deformation, assuming only a circumferential orientation of the smooth muscle.

### 4.4. Bi-layered constitutive modelling

In addition to the aforementioned data analyses, wherein medial and adventitial contributions are averaged, we conducted layer-specific equilibrium computations as previously described [14,33]. The pseudostrain energy functions and deposition stretches were specific to each constituent. For elastin, the pseudostrain energy function is represented as


(4.3) $$W^{e}\left(C^{e}\right)=\;\frac{c^{e}}{2}\left(I_{C^{e}}-3\right),$$

where $C^{e}=F^{e^{T}}F^{e}$ , and $F^{e}=FG_{h}^{e}$ where $G_{h}^{e}$ corresponds to the elastin deposition stretch tensor. For the i collagen families, the strain energy function is


(4.4) $$W^{c}\left(IV_{C}^{c,i}\right)=\frac{c_{1}^{c}}{4c_{2}^{c}}\left\{exp\left[c_{2}^{c}{\left(IV_{C}^{c,i}-1\right)}^{2}\right]-1\right\},$$

where $IV_{C}^{c,i}\;=\;{\left(G_{h}^{c,i}\right)}^{2}M^{c,i}\;\cdot \;CM^{c,i}$ , and $M^{c,i}$ relate to fibre angles as in the uni-layered case. The parameters $c_{1}^{c}$ and $c_{2}^{c}$ are assumed to be independent of collagen family, while the deposition stretches $G_{h}^{c,i}$ are collagen family specific. The total stored energy is then computed as the sum of the contributions from each constituent, where $\varphi^{e}$ , $\varphi^{c}$ and $\varphi^{m}$ represent layer-specific mass fractions of the constituents, estimated from quantitative histology, considering the fitted mass fractions of the different collagen fibre families and assuming


(4.5) $$W=\varphi^{e}W^{e}\left(C^{e}\right)\;+\;\varphi^{c}\sum_{i=1}^{4}\;\varphi^{c,i}W^{c}\left(IV_{C}^{c,i}\right)+\varphi^{m}W^{m}\left(IV_{C}^{m}\right),$$

with $\varphi^{e}$ , $\varphi^{c}$ and $\varphi^{m}$ the layer-specific mass fractions of the constituents estimated from quantitative histology and $\;\varphi^{c,i}$ are the mass fractions of the different collagen fibre families (whose sum equals 1, and $\;\varphi^{c,3}=\varphi^{c,4}$ for the two diagonal families) defined by fitting the model. Following the nonlinear regression process as described in the preceding section, the model was used to determine Cauchy stresses and material stiffness, now dependent on the radial coordinate within the medial and adventitial layers.

### 4.5. Quantitative histology

Following mechanical testing, vessels were fixed in their unloaded state in a 10% formalin solution for 24 h, then placed in a 70% ethanol solution, embedded in paraffin, sectioned (5 µm thickness), mounted and stained using Movat’s pentachrome, which stains elastin and nuclei black, collagen fibres yellowish, glycosaminoglycans blue and cell cytoplasm red. Immunohistochemical stains for CD45+, CD3+ (ab10558 and ab16669, Abcam PLC, Cambridge, UK, respectively) and CD68+ (AA312−326, antibodies-online, Aachen, Germany) cells, both at 1 : 100 with 3,3′-diaminobenzidine on a horseradish peroxidase conjugated secondary antibody, stained leucocytes (CD45+), T-cells (CD3+) and macrophages and monocytes (CD68+) brown-orange. Images were acquired using an Olympus BX/51 microscope equipped with a DP70 digital camera (effective sensor resolution of 4080 × 3072 pixels, corresponding to a pixel size of 2.1 μm at a 2/3’ sensor size) and using a 20 × objective (UPlanFl 20×, NA 0.50, optical resolution at λ = 400 nm at approx. 0.49 μm) and 0.5× tube lens, resulting in 10× magnification and hence an image resolution of 0.21 μm (fulfilling the Nyquist criterion). Images were recorded using Olympus CellSens Dimension software. When arterial cross-sections exceeded the field-of-view, multiple images were acquired and stitched using Image Composite Editor software (Microsoft Research).

### 4.6. Statistics

Most data are presented as means and standard errors and analysed using one- or two-way analysis of variance as appropriate, with post hoc Bonferroni tests. Correlation analyses between histological parameters and mechanical metrics were assessed using non-parametric Spearman correlation coefficient. Normality was checked via a Shapiro–Wilk normality test. For all reported comparisons, *p* < 0.05 was considered significant, with one-to-four asterisks denoting *p* values lower than 0.05, 0.01, 0.001 and 0.0001, respectively.

## Data Availability

Supplementary material is available online [53].
